# Characterising grey-white matter relationships in recent-onset psychosis and its association with cognitive function

**DOI:** 10.1016/j.nicl.2025.103824

**Published:** 2025-06-10

**Authors:** Yoshito Saito, Christos Pantelis, Vanessa Cropley, Liliana Laskaris, Cassandra M.J. Wannan, Warda T. Syeda

**Affiliations:** a Department of Psychiatry, The University of Melbourne, Carlton, VIC, Australia; b Western Centre for Health Research & Education, University of Melbourne & Western Health, Sunshine Hospital, St Albans, VIC, Australia; c Monash Institute of Pharmaceutical Sciences (MIPS), Monash University, Clayton, VIC, Australia; d Centre for Youth Mental Health, The University of Melbourne, Parkville, VIC, Australia; e Orygen, Parkville, VIC, Australia

**Keywords:** Schizophrenia-spectrum disorders, Recent-onset psychosis, MRI, Brain structure, Partial least squares correlation, Cognition

## Abstract

Individuals with recent-onset psychosis (ROP) present widespread grey matter (GM) reductions and white matter (WM) abnormalities. While prior studies used univariate approaches, understanding how multiple GM regions relate to WM tracts is important, as psychosis involves network-level brain dysfunction. Understanding characteristic GM-WM patterns may also clarify the basis of cognitive impairments, which are potentially linked to network dysfunction in psychosis. Using multivariate analysis, we examined whole-brain GM-WM relationships and their association with cognitive abilities in ROP.

We used T1 and diffusion-weighted images from 71 non-affective ROP individuals (age 22.09 ± 3.08) and 71 matched controls (age 22.05 ± 3.21). We performed multiblock partial least squares correlation (MB-PLS-C) to identify GM-WM patterns based on GM thickness or surface area and WM fractional anisotropy (FA), and examined their associations with cognitive abilities.

MB-PLS-C identified a ‘GM thickness’–‘WM FA’ pattern representing group differences, explaining 12.38 % of the variance and associated with frontal and temporal GM regions and seven WM tracts around subcortical structures. MB-PLS-C also identified a ‘GM surface area’–‘WM FA’ pattern showing group differences, explaining 18.92 % and related with cingulate, frontal, temporal, and parietal GM regions and 15 WM tracts, including the inferior cerebellar peduncle and corona radiata. The ‘GM thickness’–‘WM FA’ pattern describing group differences was significantly correlated with processing speed in ROP.

MB-PLS-C identified differential whole-brain GM-WM relationships, indicating a potential signature of brain alterations in ROP. Our findings of a relationship between processing speed and GM-WM patterns for GM thickness have implications for our understanding of brain-behaviour relationships in psychosis.

## Highlights

• Grey and white matter relationships compared in recent-onset psychosis and controls.
• Multiblock partial least squares correlation technique was applied.
• Both developmentally driven and disease-related GM-WM patterns were observed.
• The GM thickness-WM pattern showing group differences was linked to processing speed.

## 1 Introduction

Widespread grey matter (GM) and white matter (WM) abnormalities are observed in schizophrenia-spectrum disorders, with the greatest degree of change observed in the first 2–5 years of illness (Bartholomeusz et al., 2017, Collins et al., 2023, Kelly et al., 2018, Klauser et al., 2017, Serpa et al., 2017, van Erp et al., 2018, Zeng et al., 2016). Although these brain changes are often studied separately, recent studies suggest that abnormalities in both GM and WM are interrelated. GM volume loss follows the architecture of WM pathways and propagates along them (Chopra et al., 2023, Shafiei et al., 2020). Similarly, cortical thinning has been associated with changes in the adjacent WM regions (Di Biase et al., 2019). These findings support the hypothesis that widespread GM alterations in schizophrenia-spectrum disorders are closely associated with the underlying architecture of directly connected WM pathways. Given that the brain functions through distributed networks, GM abnormalities may also be coupled with changes in non-adjacent but functionally linked WM tracts (Avena-Koenigsberger et al., 2017a, Avena-Koenigsberger et al., 2017b, Mišić et al., 2014). Therefore, to fully understand the structural pathology of psychosis, it is important to investigate GM-WM relationships at the whole-brain level. Multivariate approaches may help clarify how widespread GM and WM abnormalities are structurally related in psychosis.

Partial least squares correlation (PLS-C) provides one possible approach to examining multivariate whole-brain GM-WM relationships (Krishnan et al., 2011, Mihalik et al., 2022). It finds key latent correlations between two sets of variables, identifying patterns overlapping between groups or representing group differences (Jessen et al., 2019, Kirschner et al., 2020, Kristensen et al., 2019, Raghava et al., 2021, Syeda et al., 2022). It is particularly effective when variables are intercorrelated, as commonly seen in neuroimaging data, and remains effective even when the number of variables exceeds the number of samples (Krishnan et al., 2011, Mihalik et al., 2022). We have developed a novel PLS-C approach, multiblock PLS-C (MB-PLS-C), which demonstrates the effects of covariates on each latent pattern (Syeda et al., 2022). MB-PLS-C therefore has the potential to identify whole-brain correlated GM-WM couplings in schizophrenia-spectrum disorders, considering covariates such as age and sex.

Investigating the relationship between whole-brain GM-WM patterns and cognitive abilities is also important for understanding cognitive impairments in schizophrenia-spectrum disorders (Green et al., 2019). A recent systematic review reported inconsistent associations between cognitive impairment and specific GM regions (Karantonis et al., 2021), and findings on WM-cognition relationships have been mixed as well (Goghari et al., 2014, Holleran et al., 2020, Joo et al., 2018, Martin et al., 2014, Nestor et al., 2013, Wannan et al., 2022). Examining GM or WM alone may not capture the full extent of neural contributions to cognitive impairment, given that cognitive functions are associated with large-scale brain networks and interactions among multiple brain regions (Bressler and Menon, 2010, Imms et al., 2021, Lara and Wallis, 2015). Therefore, examining relationships between cognition and latent variables that summarise differential GM-WM patterns may provide a more comprehensive understanding of the neural correlates of cognitive impairment.

In the current study, we aimed to 1) identify multivariate and coupled whole-brain GM-WM patterns in recent-onset psychosis (ROP) using MB-PLS-C and 2) examine how these GM-WM patterns are associated with cognitive abilities. We hypothesised that MB-PLS-C between GM and WM would identify two distinct patterns: one shared between healthy controls and ROP individuals and another that distinguishes the groups by showing opposite directions of GM-WM associations. We also hypothesised that cognitive impairments across multiple domains would be associated with the GM-WM pattern representing a group difference. Finally, we assessed the anatomical basis of these multivariate GM-WM patterns by examining structural connectivity.

## 2 Materials and methods

### 2.1 Participants

The study included 71 individuals with recent-onset psychosis (ROP) within five years of their first psychotic episode from the Human Connectome Project for Early Psychosis (HCP-EP) and 71 healthy controls (HCs) from two datasets: 34 from HCP-EP and 37 from the HCP-Development (HCP-D) (Lewandowski et al., 2020, Somerville et al., 2018). The controls were selected from healthy individuals within the HCP-EP and HCP-D using propensity score matching so that the number of participants, sex, and age were matched between the groups (Austin, 2011, Rosenbaum and Rubin, 1983). Demographic and clinical data, details of the datasets and propensity score matching are described in the Supplementary Material (see Fig. S1 and Table S1-2).

### 2.2 Cognitive assessment

Working memory, episodic memory, processing speed, and word reading ability were assessed using the NIH Cognition Toolbox (Table S3) (Gershon et al., 2013, Hodes et al., 2013, Weintraub et al., 2013).

### 2.3 MRI acquisition and processing

T1- and diffusion-weighted images were obtained from the HCP-EP and HCP-D studies within the HCP consortium, acquired using 3 T Siemens MAGNETOM Prisma scanners at Indiana University, Brigham and Women's Hospital, McLean Hospital, and Harvard University. Both studies followed equivalent imaging protocols, including T1-weighted images with 0.8 mm isotropic resolution and diffusion-weighted images (1.5 mm isotropic, TR = 3230 ms, TE = 89.20 ms) with two b-values (1500 and 3000 s/mm2), and 92 diffusion directions and 14 b0 images. Further sequence details are available in the original protocol publications (Lewandowski et al., 2020, Somerville et al., 2018).T1-weighted images were processed by the HCP consortium, and we processed the diffusion-weighted images with the HCP minimal preprocessing pipeline (Glasser et al., 2013). Thickness, surface area, and volume of GM regions were calculated using FreeSurfer (v6.0.0) (https://surfer.nmr.mgh.harvard.edu) according to the Desikan-Killiany Atlas (Desikan et al., 2006). Fractional anisotropy (FA) was computed using tract-based spatial statistics analysis with FSL (v6.0.4) for WM tracts of Johns Hopkins University white matter tract atlas (Mori et al., 2005). Harmonisation using ComBat was applied to GM and WM variables to reduce the effect of the scanner and protocol differences (Fortin et al., 2018, Fortin et al., 2017, Johnson et al., 2007). Further details are provided in the Supplementary Material (see Fig. S2-4 and Tables S4-5).

### 2.4 Statistical analyses

Demographic, clinical, cognitive, and GM and WM measures were compared between groups using the Mann-Whitney *U* test, chi-square test, or analysis of covariance based on data type and covariates. Correlations were assessed using Pearson’s or Spearman’s correlation coefficient based on the normality of the data.

#### 2.4.1 Multiblock partial least squares correlation analyses (MB-PLS-C)

**Fig. 1:**
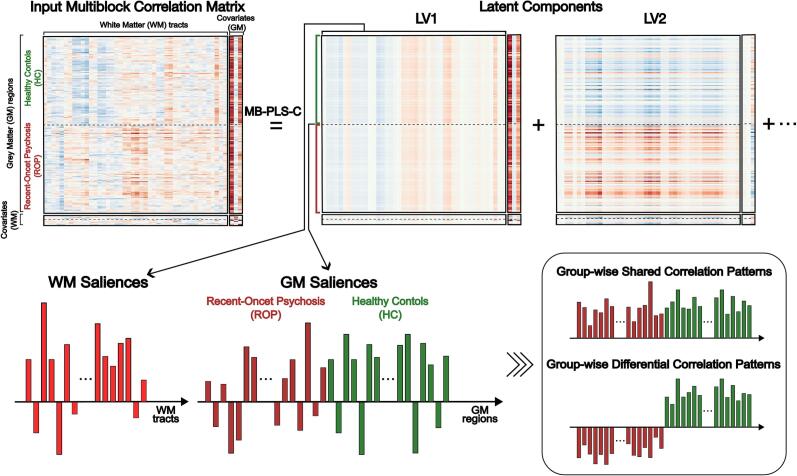
MB-PLS-C and interpretation of GM and WM saliences. Key: An input multiblock correlation matrix between GM and WM variables with their covariates is decomposed into latent components using singular value decomposition. The latent components described a pattern of features (referred to as ‘salience’ in this paper) which represents the relevance of GM regions and WM tracts to each latent variable.

This study employed MB-PLS-C to identify multivariate GM-WM patterns (Fig. 1). MB-PLS-C identifies latent variables (LVs) that maximise covariance between two multivariate data blocks and between data and covariate blocks (Syeda et al., 2022). Details of MB-PLS-C are in the Supplementary Material. Briefly, a multiblock correlation matrix was formed by combining the correlation matrices of GM and WM variables with their covariates. The matrix was created for each group and stacked vertically. This matrix was decomposed into singular vector matrices, representing the degree to which each GM or WM variable is related to the latent variables. These values, referred to as ‘saliences’ in the PLS literature, indicate how strongly each variable contributes to the identified multivariate pattern (Mihalik et al., 2022). Permutation tests assessed the significance of the overall pattern and LVs with 10,000 permutations, and the confidence interval for saliences was estimated by bootstrapping with 10,000 iterations. Model generalisability was assessed by cross-validation and an out-of-sample analysis.

#### 2.4.2 Interpretation of saliences

The salience pattern of each LV illustrates the contribution of GM and WM variables to their corresponding LV (Fig. 1). GM saliences describe group-wise patterns, where the same polarity indicates shared correlation patterns, while the opposite polarity reflects group-differentiating patterns. Saliences of WM variables represent an overall pattern averaged across the groups. This difference in the expression of GM and WM saliences arises from the way the multiblock matrix was constructed, where we investigated differential patterns in GM structures in relation to the WM tracts (Fig. 1). The product of GM and WM saliences reflects the strength of a GM-WM pattern, and the polarity of the product suggests the direction of the pattern.

#### 2.4.3 Further analysis of MB-PLS-C results

Group differences in GM saliences were evaluated by the normalised between-group structural salience difference (NSSD) for each LV (Syeda et al., 2022). To examine the convergence of salience values with increasing sample size, we calculated the absolute difference in each salience between N + 1 and N samples and plotted trajectories. Please refer to the Supplementary Material for further details.

#### 2.4.4 Tractography-based validation of GM-WM patterns derived from MB-PLS-C

Tractography was performed to assess the anatomical basis of GM-WM patterns describing group differences (see Fig. S10). Whole-brain tractography was performed using multi-shell, multi-tissue constrained spherical deconvolution to estimate fibre orientation distributions (FODs), followed by iFOD2 probabilistic tracking with anatomically constrained tractography, generating 10 million streamlines per subject (Dhollander et al., 2019, Jeurissen et al., 2014, Smith et al., 2012, Tournier et al., 2010). Streamline weights were refined using SIFT2 (Smith et al., 2015). Streamlines connecting significant GM regions and passing through significant WM tracts defined by the DK and JHU atlases in the GM-WM patterns were extracted from the whole-brain tractogram, and their total weight was calculated. To assess if the connectivity of the GM-WM pattern was significantly high, we generated 10,000 random GM-WM patterns, each consisting of the same number of GM regions and WM tracts as the original pattern, for comparison. More details are in the Supplementary Material.

#### 2.4.5 Correlation between cognitive abilities and LVs of GM-WM patterns showing group differences

Each participant was assigned a latent variable in MB-PLS-C, indicating how strongly they expressed the corresponding GM-WM pattern. To investigate the relationship between cognitive performance and these brain patterns showing group differences, correlations between each cognitive ability and the latent variable were evaluated in ROP individuals and controls separately. Multiple comparison corrections were applied using the Benjamini-Hochberg (BH) method with a 5 % false discovery rate ([4 cognitive abilities] × [GM or WM latent variables]).

#### 2.4.6 Follow-up analyses

Correlations between latent variables and three PANSS scores (positive, negative, and general psychopathology scales) and antipsychotic doses were evaluated. By using allometric scaling maps, we evaluated the similarity between cortical variations based on typical development and the GM patterns identified through MB-PLS-C analysis (Reardon et al., 2018). Details are described in the Supplementary Material.

## 3 Results

### 3.1 Clinical and demographic characteristics of the participants

ROP and HC groups were matched in sex (ROP: 69.01 % male; HC: 70.42 % male) and age (ROP: 22.09 ± 3.08 years; HC: 22.05 ± 3.21 years) (Table S1). Within the ROP group, primary lifetime diagnoses included schizophrenia (71.8 %), schizoaffective disorder (12.7 %), schizophreniform disorder (11.3 %), and other non-affective psychotic disorders (4.2 %).

### 3.2 Case-control differences in cognition, GM, and WM

ROP individuals showed significantly poorer performance for all the cognitive measures (Table S7). GM thickness showed widespread reduction, primarily in the temporal, parietal, and frontal lobes and insula (Fig. S6). No between-group differences were observed in GM surface area, as well as in FA of any WM tracts (Fig. S7).

### 3.3 MB-PLS-C between GM thickness and WM FA

The MB-PLS-C analyses between GM thickness and WM FA were significant (omnibus test *p* < 0.0005). While LV1 was not significant, LV2 and LV3 were significant and explained 29.30 % of sum-of-squares variance (Table 1, Fig. S8, and Table S8).

**Table 1:** Results of the MB-PLS-C analysis.

Latent variable	Sum-of-squares covariance, ξ_i_* (%)	*p*	Block-wise Contribution to Variance (σ_i_*)	Key GM Regions	WM Tracts	NSSD Highlights
LV2 (GM Cortical Thickness-WM FA)	16.92	0.04	GM-WM: 66.36 % WM-WMcov: 1.28 % GM-GMcov: 28.24 %	HC: rh pars opercularis rh superior frontal lh superior frontal rh pars triangularis lh precuneus ROP: rh rostral anterior cingulate rh para hippocampal rh posterior cingulate lh cuneus rh isthmus cingulate	r hippocampal gyrus part of cingulum l hippocampal gyrus part of cingulum l medial lemniscus r cingulate gyrus part of cingulum l cingulate gyrus part of cingulum r cerebral peduncle l cerebral peduncle l sagittal stratum r posterior thalamic radiation r anterior corona radiata	HC > ROP: rh pars opercularis rh superior frontal rh pars triangularis lh pars triangularis lh posterior cingulate
LV3 (GM Cortical Thickness-WM FA)	12.38	0.003	GM-WM: 84.22 % WM-WMcov: 3.86 % GM-GMcov: 9.41 %	HC: lh inferior temporal lh pericalcarine rh temporal pole lh entorhinal lh fusiform ROP: lh medial orbitofrontal lh fusiform lh frontal pole lh rostral middle frontal lh superior frontal	r corticospinal tract r fornix stria terminalis l anterior limb of internal capsule r anterior limb of internal capsule l posterior thalamic radiation l retrolenticular part of internal capsule r retrolenticular part of internal capsule	ROP > HC: lh medial orbitofrontal lh superior frontal lh fusiform lh rostralanterior cingulate HC > ROP: lh cuneus
LV1 (GM Surface Area-WM FA)	53.21	0.013	GM-WM: 29.67 % WM-WMcov: 0.61 % GM-GMcov: 67.86 %	HC: rh middle temporal rh precuneus lh superior temporal lh middle temporal rh superior temporal ROP: rh middle temporal lh inferior temporal lh superior frontal rh superior frontal lh middle temporal	r uncinate fasciculus l superior corona radiata l external capsule splenium of corpus callosum r anterior limb of internal capsule r external capsule r anterior corona radiata r superior corona radiata l anterior corona radiata	HC > ROP: lh entorhinal rh transverse temporal rh entorhinal rh superior temporal rh pars orbitalis
LV2 (GM Surface Area-WM FA)	18.97	0.0003	GM-WM: 91.13 % WM-WMcov: 6.81 % GM-GMcov: 1.86 %	HC: rh pars opercularis lh entorhinal rh isthmus cingulate rh caudal middle frontal lh superior parietal ROP: lh paracentral lh caudal middle frontal rh entorhinal rh postcentral lh caudal anterior cingulate	l inferior cerebellar peduncle l posterior corona radiata l superior corona radiata l superior longitudinal fasciculus r posterior corona radiata r inferior cerebellar peduncle l sagittal stratum r posterior limb of internal capsule r sagittal stratum r superior corona radiata r superior longitudinal fasciculus l anterior limb of internal capsule r medial lemniscus l posterior limb of internal capsule r corticospinal tract r external capsule	ROP > HC: rh superior parietal lh precentral rh supramarginal HC > ROP: rh temporal pole lh superior parietal

Key: For each latent variable, the table presents the percentage of sum-of-squares cross-block covariance (ξ_i_), *p*-value, and block-wise contributions of each latent variable to the singular value (σ_i_). It also summarises the top five GM regions with the highest salience (absolute value) per group, significant WM tracts, and group differences in GM saliences based on NSSD.

*σ_i_ and ξ_i_ represent the singular values and the percentage of the cross-block covariance explained by the ith significant latent variable, i ∈ {2, 3 (cortical thickness); 1, 2 (cortical surface area)}.

#### 3.3.1 Second latent variable (LV2)

**Fig. 2:**
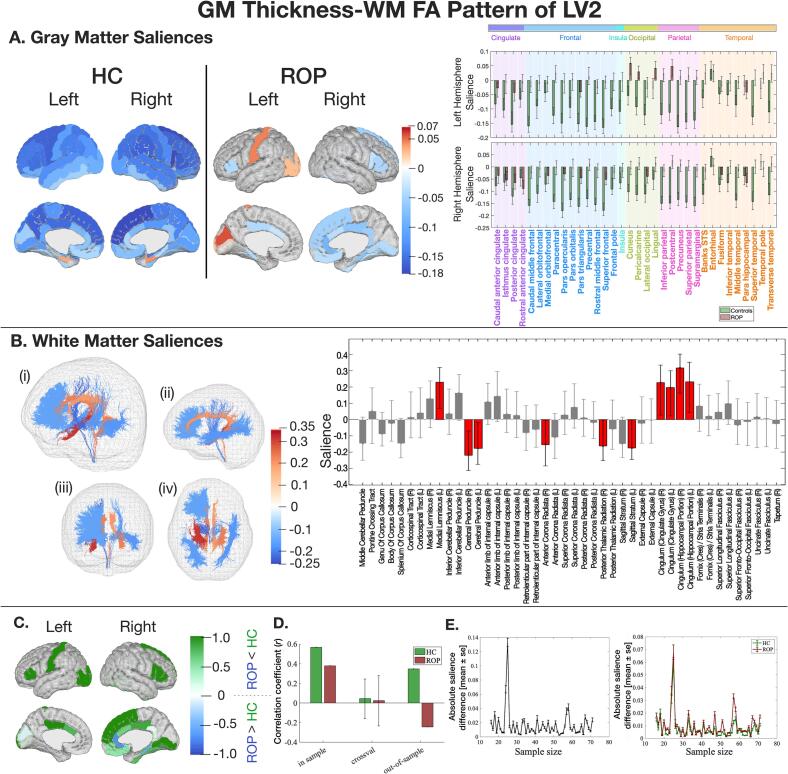
The latent pattern of LV2 between GM thickness and WM FA. Key: A) GM saliences by group in HC (green) and ROP (red) with 95 % confidence interval and color-coded lobe information. B) WM saliences shared between groups. WM tracts are colour-coded based on the salience intensity ((i) overall view, (ii) from the left, (iii) front, and (iv) bottom). The bar plot shows the salience of each WM tract with 95 % confidence intervals. C) Normalised structural salience-differences (NSSDs). A positive score (green) shows a salience is larger in HC, and a negative score (blue) shows a salience is larger in ROP. D) A correlation coefficient between LV2 of GM and WM in the training samples (in sample) and the test samples (out-of-sample), and a correlation coefficient and its standard deviation from Monte Carlo cross-validation. E) The impact of sample size on salience intensity (left: WM saliences, right: GM saliences). (For interpretation of the references to color in this figure legend, the reader is referred to the web version of this article.)

LV2 reflected a negative correlation between age and GM thickness in the covariate blocks in controls (Fig. S8). Almost all significant GM saliences in controls were consistently large and in the same direction, while those in the ROP group were more variable, sparse, and smaller (Fig. 2A). Key GM regions with the highest salience and significant WM tracts are listed in Table 1.

In the training sample (n = 142), both groups showed a positive correlation between latent variables of GM thickness and WM FA (HC: *r* = 0.57, p < 0.001, ROP: *r* = 0.38, *p* = 0.001). A significant correlation was not observed in the cross-validation data (n = 112 in the training set, n = 30 in the test set), and only controls showed a generalisable correlation in the out-of-sample results (n = 51) (Fig. 2D). GM and WM saliences showed convergence when the sample size reached 25 samples per group (Fig. 2E).

#### 3.3.2 Third latent variable (LV3)

**Fig. 3:**
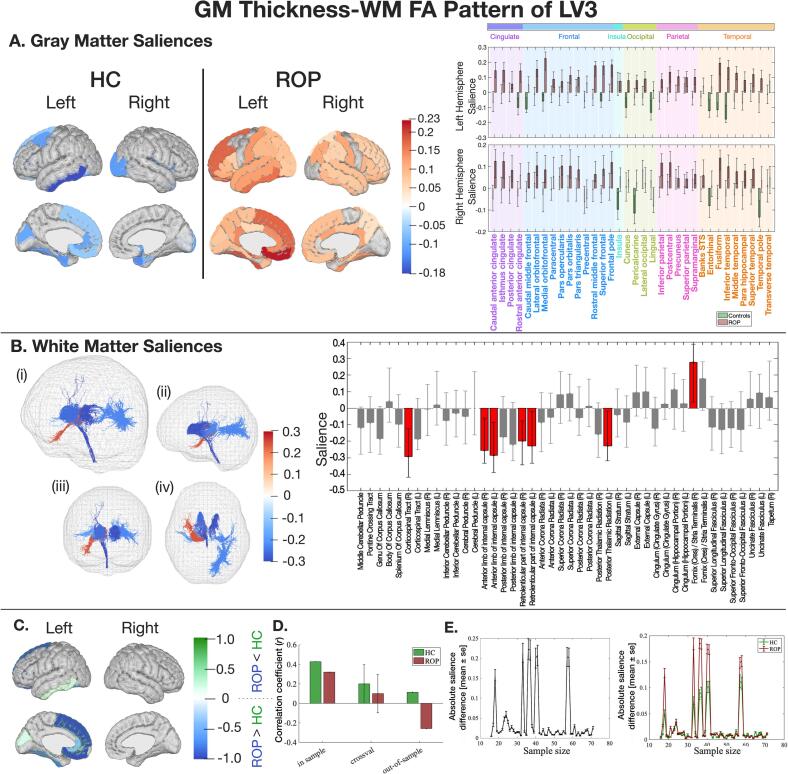
The latent pattern of LV3 between GM thickness and WM FA. Key: A) GM saliences. B) WM saliences. C) NSSDs. D) A correlation coefficient between LV3 of GM and WM, with Monte Carlo cross-validation results. E) The impact of sample size on salience intensity. Please refer to Fig. 2 for detailed explanations.

In LV3, ROP individuals demonstrated negative correlations between GM thickness and WM FA, whereas controls demonstrated positive correlations (Fig. S8). A GM salience pattern described group differences, where saliences were strongly mapped onto the ROP group while controls showed smaller saliences in the other direction (Fig. 3A). Key GM regions and WM tracts with a significant salience are listed in Table 1. In the training sample (n’s as above), both groups showed a positive correlation between latent variables of GM thickness and WM FA (HC: *r* = 0.43, *p* < 0.001, ROP: *r* = 0.32, *p* = 0.007). The HC group only demonstrated a significant correlation in cross-validation data (n’s as above), and a generalisable correlation was not demonstrated in the out-of-sample data (n’s as above) (Fig. 3D). GM and WM saliences showed convergence when the sample size reached 60 per group (Fig. 3E).

### 3.4 MB-PLS-C between GM surface area and WM FA

The MB-PLS-C analyses between GM surface area and WM FA were significant (omnibus test *p* < 0.0005). LV1 and LV2 were significant and explained 72.18 % of sum-of-squares variance (Table 1, Fig. S9, and Table S9).

#### 3.4.1 First latent variable (LV1)

**Fig. 4:**
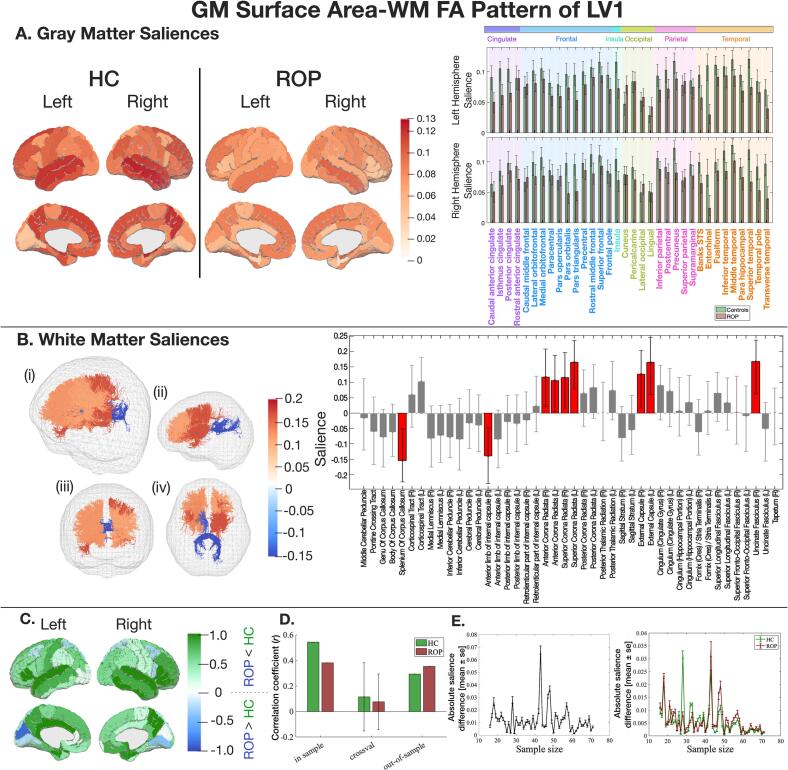
The latent pattern of LV1 between GM surface area and WM FA. Key: A) GM saliences. B) WM saliences. C) NSSDs. D) A correlation coefficient between LV1 of GM and WM, with Monte Carlo cross-validation results. E) The impact of sample size on salience intensity. Please refer to Fig. 2 for detailed explanations.

Both groups showed a similar correlation pattern. The covariate blocks displayed a robust positive correlation between increased GM surface areas and increased total intracranial volume, which was also associated with male sex (Fig. S9). Significant GM saliences showed a pattern overlapping between the groups, with the controls demonstrating a stronger mapping than the ROP group (Fig. 4A). Key GM regions and WM tracts with a significant salience are listed in Table 1. In the training sample (n’s as above), both groups showed a positive correlation between latent variables of GM surface area and WM FA (HC: *r* = 0.54, *p* < 0.001, ROP: *r* = 0.38, *p* = 0.001). Whilst both groups did not show significant correlations in cross-validation data (n’s as above), out-of-sample data (n’s as above) displayed similar correlations in both groups (Fig. 4D). GM and WM saliences exhibited convergence when the sample size reached 50 per group (Fig. 4E).

#### 3.4.2 Second latent variable (LV2)

**Fig. 5:**
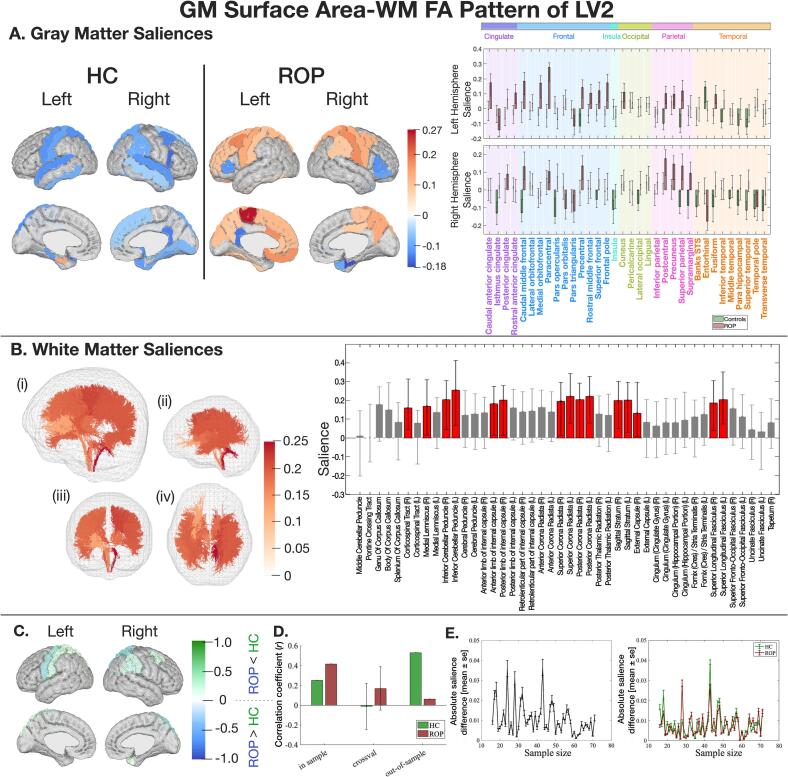
The latent pattern of LV2 between GM surface area and WM FA. Key: A) GM saliences. B) WM saliences. C) NSSDs. D) A correlation coefficient between LV2 of GM and WM, with Monte Carlo cross-validation results. E) The impact of sample size on salience intensity. Please refer to Fig. 2 for detailed explanations.

The HC group demonstrated negative correlations between GM surface area and WM FA, while the ROP group exhibited positive correlations (Fig. S9). Significant GM saliences were in opposite directions between the groups, describing group differences (Fig. 5A). Key GM regions and WM tracts with a significant salience are listed in Table 1. In the training sample (n’s as above), both groups showed positive correlations between each of the latent variables for GM surface area and WM FA (HC: *r* = 0.25, *p =* 0.033, ROP: *r* = 0.42, *p* < 0.001), but there were no significant correlations observed in the cross-validation data (n’s as above). Out-of-sample data (n’s as above) demonstrated a generalised correlation only in the HC group but not in the ROP group (Fig. 5D). GM and WM saliences converged after 50 samples per group (Fig. 5E).

### 3.5 Tractography-based examination of GM-WM patterns derived from MB-PLS-C

To assess the anatomical basis of GM-WM patterns showing group differences, we calculated the strength of direct cortico-cortical connectivity within the GM-WM patterns using tractography (Fig. S10). The permutation test demonstrated significantly high direct cortico-cortical connectivity in the ‘GM surface area’–‘WM FA’ pattern (LV2) (*p* = 0.0002 (ROP), *p* = 0.012 (HC)) but not in the ‘GM thickness’–‘WM FA’ pattern (LV3) (*p* = 0.87 (ROP), *p* = 0.72 (HC)). Fig. S10B shows that the ‘GM thickness’–‘WM FA’ pattern had relatively high cortico-subcortical connectivity compared with cortico-cortical connectivity.

### 3.6 Correlations between cognitive abilities and latent variables of GM-WM patterns describing group differences

In the ‘GM thickness’–‘WM FA’ pattern demonstrating group differences (LV3), the ROP group showed a significant correlation between the WM latent variables and processing speed (*ρ* = 0.32, *p* = 0.006, *p_adjusted_* = 0.048). The HC group did not exhibit a significant correlation. The Fisher’s z-test further demonstrated a significant group difference in a correlation between the WM latent variables and processing speed (z = 2.34, *p* = 0.019). The differential ‘GM surface area’–‘WM FA’ pattern (LV2) did not show any significant correlations. The details are provided in the Supplementary Material.

### 3.7 Follow-up analyses

The latent variables derived from MB-PLS-C were not significantly correlated with PANSS scores and doses of antipsychotics. The above four GM salience patterns demonstrated significant correlations with the allometric scaling maps, except for the left hemisphere pattern in LV2 of the ROP group between GM surface area and WM FA. Details are described in the Supplementary Material.

## 4 Discussion

This study examines 1) multivariate grey matter-white matter (GM-WM) patterns in recent-onset psychosis (ROP) individuals and healthy controls (HC) using multiblock partial least squares correlation (MB-PLS-C) and 2) associations between these patterns and cognitive abilities. For cortical thickness, one latent variable (LV) showed a GM-WM coupling pattern predominantly expressed in the HC group, while the other LV captured a pattern that distinguishes the groups by showing opposite directions of GM-WM associations. For surface area, one LV exhibited a pattern shared across groups, whereas the other differentiated the groups through opposing GM-WM association patterns. Processing speed was significantly correlated with the WM latent variable of the differential ‘GM thickness’–‘WM FA’ pattern in the ROP group. These findings suggest potential signatures of aberrant GM-WM couplings in early psychosis, with an association with cognitive abilities.

### 4.1 Multivariate GM-WM coupling patterns in ROP identified by MB-PLS-C

An important advantage of MB-PLS-C is that it captures multivariate patterns of covariation between GM and WM. In contrast to univariate methods that examine GM and WM independently, this approach identifies whole-brain GM-WM covariance patterns that may better reflect the distributed brain network disruptions in schizophrenia-spectrum disorders (Fornito and Bullmore, 2015, Friston, 1998, Gao et al., 2023). Moreover, the differential GM-WM coupling patterns suggest a different configuration of GM-WM coupling between groups, potentially reflecting compensatory mechanisms, pathological processes, or neurodevelopmental deviations in the disorders (Cetin-Karayumak et al., 2020, Dandash et al., 2014, Di Biase et al., 2019).

The pattern where the association between GM thickness and WM FA was reversed between groups (LV3, 12.4 % variance) involved frontal and temporal GM regions, which are consistently implicated in schizophrenia (Bartholomeusz et al., 2017, Collins et al., 2023, van Erp et al., 2018). The associated WM tracts, including the corticospinal tract, internal capsule, and posterior thalamic radiation, are indicative of key pathways linked to cortical thinning, which have not been identified in previous univariate analyses (Di Biase et al., 2017, Filippi et al., 2014, Keymer-Gausset et al., 2018, Van Dyken et al., 2024). These findings suggest that these WM tracts function under different configurations of GM regions, as indicated by the GM patterns. Notably, these tracts are located around subcortical structures, suggesting that WM connections involving subcortical regions may contribute to cortical thinning in psychosis, consistent with cortico-subcortical dysconnectivity hypotheses (Anticevic et al., 2014, Dandash et al., 2017, Woodward et al., 2012).

The ‘GM surface area’–‘WM FA’ pattern exhibiting differential GM-WM relationships between groups (LV2, 19.0 % variance) involved frontal and parietal GM regions not identified by prior univariate surface area studies in early psychosis (Chung et al., 2019, Jalbrzikowski et al., 2019). These GM regions show longitudinal surface area contractions after psychosis onset, indicating the associated WM tracts in this pattern may contribute to this surface area contraction (Sun et al., 2009). This pattern may also reflect a developmental deviation in GM-WM coupling in schizophrenia-spectrum disorders, as it involves the superior longitudinal fasciculus and sagittal stratum, which reach their structural maturity earlier than in typically developing individuals (Cetin-Karayumak et al., 2020). Furthermore, MB-PLS-C identified an association between cortical surface area and the inferior cerebellar peduncle, which conveys proprioceptive sensory input to the cerebellum. Although not directly connected to the cortex, this tract contributed most to the pattern, suggesting an indirect influence on surface area via pathways involved in sensory prediction deficits in schizophrenia-spectrum disorders (Abram et al., 2022, Andreasen and Pierson, 2008, Sterzer et al., 2018).

Different findings for cortical thickness versus surface area suggest that each reflects distinct genetic, environmental, and neurodevelopmental aspects of the GM-WM relationship. In schizophrenia-spectrum disorders, cortical surface area is largely influenced by genetic factors and established during early stages of development, whereas cortical thickness is more sensitive to environmental factors such as neuroinflammation, stress, and substance abuse (Cannon et al., 2015, Neilson et al., 2017, van Erp et al., 2018). Notably, the WM tracts associated with the differential surface area pattern show neurodevelopmental abnormalities, while the internal capsule and thalamic radiations, which are part of the differential thickness pattern, are affected by substance abuse and smoking (Cetin-Karayumak et al., 2020, James et al., 2011, Peters et al., 2009, Zhang et al., 2010). Thus, these distinctions in GM-WM coupling may underlie different mechanisms of structural vulnerability in schizophrenia-spectrum disorders.

Another key advantage of using PLS-C is its ability to capture multiregional and reciprocal GM-WM associations that are not constrained by direct structural connectivity, in contrast to previous studies (Chopra et al., 2023, Di Biase et al., 2019, Shafiei et al., 2020). We identified an association between cortical surface area and the inferior cerebellar peduncle, a tract with no direct cortical connections. We also found that, despite the presence of a significant GM-WM pattern, tractography showed limited structural connectivity between the involved regions, indicating that this pattern may not rely on direct anatomical connections. These findings highlight MB-PLS-C’s capacity to identify key GM-WM associations reflecting network-mediated pathways in schizophrenia-spectrum disorders—conditions characterised by distributed network disruptions involving the cortex, thalamus, striatum, and cerebellum (Andreasen et al., 1998, Anticevic et al., 2014, Bernard et al., 2017, Cao et al., 2018).

### 4.2 Association between GM-WM relationships and cognitive abilities

In ROP individuals, the WM component of 3rd latent variable (the ‘GM thickness’–‘WM FA’ pattern exhibiting group differences) was significantly correlated with processing speed. Processing speed, a core cognitive deficit, is associated with WM microstructure changes (Bosma et al., 2023, Dickinson et al., 2007, Karbasforoushan et al., 2015, Klauser et al., 2021, Kochunov et al., 2017, Schaefer et al., 2013, Seitz-Holland et al., 2022). Our results extend previous findings by showing that WM alterations around subcortical structures, associated with GM thickness, are linked to processing speed in early psychosis, consistent with the role of subcortical regions in bradyphrenia (Pantelis et al., 1992). Given the rapid cortical thinning observed in the early stage, this suggests the WM alterations linked to GM thickness changes around the onset may contribute to lower processing speed (Bartholomeusz et al., 2017, Chung et al., 2019, Collins et al., 2023, Di Biase et al., 2019). As disruptions in WM hubs are linked to slower processing speed, thalamus and striatum may act as hubs where WM alterations associated with cortical thinning accumulate (Klauser et al., 2021). However, longitudinal studies are needed to assess such a causal relationship between these WM changes and processing speed.

### 4.3 Limitations and conclusion

Our study has several limitations. The antipsychotic medication effect on GM and WM changes was not accounted for. While no significant correlations between latent variables and medication doses were observed, studies with antipsychotic-naive individuals are needed. Additionally, confounders, such as substance use, illness duration, and comorbid mental and physical illnesses, were not considered due to data limitations. This study employed the MB-PLS-C analysis with a large number of variables to select only significant components, following practices in previous research. While the cross-validation and out-of-sample analysis did not confirm the generalisability of the models, results may reflect the heterogeneity in schizophrenia-spectrum disorders population, especially in early psychosis (Alnæs et al., 2019, Baldwin et al., 2022, Dwyer et al., 2023, Lv et al., 2021). Further studies with larger or diverse samples are needed to explore this heterogeneity.

In summary, our MB-PLS-C approach identified distinct whole-brain GM-WM coupling patterns between ROP individuals and controls, highlighting the value of multivariate analysis for capturing network-level brain alterations. The ‘GM thickness’–‘WM FA’ pattern showing group differences exhibited the most pronounced contribution in frontal and temporal regions and WM tracts around subcortical structures. For the ‘GM surface area’–‘WM FA’ pattern representing group differences, the most significant contribution was observed in cingulate, frontal, temporal, and parietal regions and WM tracts, such as the inferior cerebellar peduncle and superior corona radiata. The ‘GM thickness’–‘WM FA’ pattern was significantly associated with processing speed in ROP. These findings have implications for our understanding of brain-behaviour relationships in psychosis. Further studies on ultra-high-risk and chronic schizophrenia individuals are required to track latent GM-WM patterns over illness progression. Identifying these patterns and their association with cognitive abilities could inform interventions for cognitive impairments.

## Code availability

MB-PLS-C was performed in Matlab 2022b. Codes can be available from the corresponding author upon reasonable request.

## Declaration of Generative AI and AI-assisted technologies in the writing process

During the preparation of this work, the authors used ChatGPT to improve the manuscript's readability and language. After using this tool, the authors reviewed and edited the content as needed and took full responsibility for the content of the published article.

## CRediT authorship contribution statement

**Yoshito Saito:** Writing – review & editing, Writing – original draft, Visualization, Validation, Software, Methodology, Investigation, Formal analysis, Data curation, Conceptualization. **Christos Pantelis:** Writing – review & editing, Supervision, Resources, Methodology, Funding acquisition, Conceptualization. **Vanessa Cropley:** Writing – review & editing, Investigation, Funding acquisition, Data curation. **Liliana Laskaris:** Writing – review & editing, Investigation, Data curation. **Cassandra M.J. Wannan:** Writing – review & editing, Supervision, Methodology, Conceptualization. **Warda T. Syeda:** Writing – review & editing, Supervision, Software, Methodology, Conceptualization.

## Declaration of competing interest

The authors declare that they have no known competing financial interests or personal relationships that could have appeared to influence the work reported in this paper.

## Data Availability

Brain imaging and cognitive and clinical data from the Human Connectome Project for early psychosis can be requested from https://www.humanconnectome.org/study/human-connectome-project-for-early-psychosis. Brain imaging and cognitive data from the Human Connectome Project Development can be requested from https://www.humanconnectome.org/study/hcp-lifespan-development. Access to these datasets requires approval. Other data can be obtained from the corresponding author upon reasonable request.
